# Improving Clinical Translation of Machine Learning Approaches Through Clinician-Tailored Visual Displays of Black Box Algorithms: Development and Validation

**DOI:** 10.2196/15791

**Published:** 2020-06-09

**Authors:** Shannon Wongvibulsin, Katherine C Wu, Scott L Zeger

**Affiliations:** 1 Department of Biomedical Engineering Johns Hopkins University School of Medicine Baltimore, MD United States; 2 Department of Medicine Division of Cardiology Johns Hopkins University School of Medicine Baltimore, MD United States; 3 Department of Biostatistics Johns Hopkins Bloomberg School of Public Health Baltimore, MD United States

**Keywords:** machine learning, interpretability, clinical translation, prediction models, visualization

## Abstract

**Background:**

Despite the promise of machine learning (ML) to inform individualized medical care, the clinical utility of ML in medicine has been limited by the minimal interpretability and *black box* nature of these algorithms.

**Objective:**

The study aimed to demonstrate a general and simple framework for generating clinically relevant and interpretable visualizations of *black box* predictions to aid in the clinical translation of ML.

**Methods:**

To obtain improved transparency of ML, simplified models and visual displays can be generated using common methods from clinical practice such as decision trees and effect plots. We illustrated the approach based on postprocessing of ML predictions, in this case random forest predictions, and applied the method to data from the Left Ventricular (LV) Structural Predictors of Sudden Cardiac Death (SCD) Registry for individualized risk prediction of SCD, a leading cause of death.

**Results:**

With the LV Structural Predictors of SCD Registry data, SCD risk predictions are obtained from a random forest algorithm that identifies the most important predictors, nonlinearities, and interactions among a large number of variables while naturally accounting for missing data. The *black box* predictions are postprocessed using classification and regression trees into a clinically relevant and interpretable visualization. The method also quantifies the relative importance of an individual or a combination of predictors. Several risk factors (heart failure hospitalization, cardiac magnetic resonance imaging indices, and serum concentration of systemic inflammation) can be clearly visualized as branch points of a decision tree to discriminate between low-, intermediate-, and high-risk patients.

**Conclusions:**

Through a clinically important example, we illustrate a general and simple approach to increase the clinical translation of ML through clinician-tailored visual displays of results from black box algorithms. We illustrate this general model-agnostic framework by applying it to SCD risk prediction. Although we illustrate the methods using SCD prediction with random forest, the methods presented are applicable more broadly to improving the clinical translation of ML, regardless of the specific ML algorithm or clinical application. As any trained predictive model can be summarized in this manner to a prespecified level of precision, we encourage the use of simplified visual displays as an adjunct to the complex predictive model. Overall, this framework can allow clinicians to peek inside the black box and develop a deeper understanding of the most important features from a model to gain trust in the predictions and confidence in applying them to clinical care.

## Introduction

### Background

There is growing interest in benefiting from the predictive power of machine learning (ML) to improve the outcomes of medical care at more affordable costs. Although notable for their impressive predictive ability, ML *black box* predictions are often characterized by minimal interpretability, limiting their clinical adoption despite their promise for improving health care [1-6]. As a result, there is growing emphasis on the field of *interpretable ML* or *explainable Artificial Intelligence* to provide explanations of how models make their decisions [6-8]. However, the lack of understanding of how ML predictions are generated and the complex relationships between the predictors and outcomes are still obstacles to the adoption of ML in clinical practice.

Many approaches have been developed to explain predictions and determine ML feature importance and effect, but they have limited adoption in real-world clinical applications [9-12]. There have been previous proposals to stack ML methods or to use rule extraction with ML output to produce simpler summaries, but because of their inherent complexities or lack of clinical applications, these tools are seldom used in medicine [13-16].

### Objectives

To accelerate the integration of ML into clinical care, an emphasis on the personalization of these tools for the end user is crucial. Our work is motivated by the well-known clinical challenge of measuring an individual's risk of sudden cardiac death (SCD), a leading cause of death with inherently complex pathophysiology that lends itself to novel approaches [17-22]. Although we focus here on SCD as an illustrative example, the methods we present are applicable more broadly to improving clinical translation of ML, regardless of the specific ML algorithm or clinical application. The contribution of this work is a general framework for translating complex *black box* predictions into easily understood representations through commonly encountered clinical summaries. Overall, we emphasize the need for multidisciplinary teams to create clinician-tailored visual displays that provide interpretability in ways that are personalized to the clinician’s preferences for understanding ML predictions to aid in effective clinical translation of ML.

## Methods

### Data Source

The Left Ventricular (LV) Structural Predictors of SCD Registry is a prospective observational registry (clinicaltrials.gov, NCT01076660), which enrolled 382 patients for the primary end point of an adjudicated appropriate implantable cardioverter defibrillator firing for ventricular tachycardia or ventricular fibrillation or SCD not aborted by the device [23-29]. In the 8-year follow-up, 75 individuals had the primary outcome.

### Modeling

Our ML approach is based on the random forest (RF) algorithm implemented in the randomForestSRC R package [30] extended to time-varying SCD risk prediction [31]. RF is an ensemble learning method based on a collection of decision trees, where the overall RF prediction is the ensemble average or majority vote. Random sampling of predictor variables at each decision tree node and bootstrapping the original training data decrease the correlation among the trees in the forest to allow for impressive predictive performance [32,33]. For our RF, the predictors included demographics, comorbidities, medications, electrophysiologic parameters, laboratory values, LV ejection fraction by echocardiography, and cardiac magnetic resonance (CMR) imaging indices, summarized in Table 1.

**Table 1:** Patient characteristics in the Left Ventricular Structural Predictors of Sudden Cardiac Death Registry (N=382).

Variables			No. of SCD^a^ event (n=307)		Patient with SCD event (n=75)		*P* value^b^	
**Demographics and clinical characteristics**								
	Age (years), mean (SD)			57 (13)		57 (12)		.75
	Male, n (%)			211 (68.7)		63 (84)		.*01*
	**Race, n (%)**							**.66**
		White	200 (65.1)		51 (68)			
		African American	99 (32)		21 (28)			
		Other	8 (3)		3 (4)			
	Body surface area (m^2^), mean (SD)			1.98 (0.28)		2.05 (0.28)		.07
	Ischemic cardiomyopathy etiology, n (%)			149 (48.5)		44 (59)		.15
	Years from incident MI^c^ or cardiomyopathy diagnosis, mean (SD)			3.83 (5.18)		5.43 (5.61)		*.02*
	**NYHA^d^ functional class, n (%)**							**.55**
		I	64 (21)		20 (27)			
		II	137 (44.6)		31 (41)			
		III	106 (34.5)		24 (32)			
	One or more heart failure hospitalizations, n (%)			0 (0)		19 (25.3)		*<.001*
**Cardiac risk factors, n (%)**								
	Hypertension			180 (58.6)		44 (59)		>.99
	Hypercholesterolemia			180 (58.6)		45 (60)		.93
	Diabetes			85 (28)		19 (25)		.79
	Nicotine use			133 (43.3)		44 (59)		*.02*
**Medication usage, n (%)**								
	ACE^e^-inhibitor or ARB^f^			275 (89.6)		66 (88)		.85
	Beta-blocker			288 (93.8)		68 (91)		.48
	Lipid-lowering			199 (64.8)		56 (75)		.14
	Antiarrhythmics (amiodarone)			18 (6)		8 (11)		.22
	Diuretics			173 (56.4)		54 (72)		*.02*
	Digoxin			50 (16)		16 (21)		.39
	Aldosterone inhibitor			80 (26)		21 (28)		.85
	Aspirin			215 (70.0)		55 (73)		.67
**Electrophysiologic variables**								
	Prior atrial fibrillation, n (%)			51 (17)		14 (19)		.80
	Ventricular rate (bpm), mean (SD)			73 (14)		70 (14)		.06
	QRS duration (ms), mean (SD)			118 (31)		122 (27)		.30
	Presence of LBBB^g^, n (%)			79 (26)		14 (19)		.26
	Biventricular ICD^h^, n (%)			90 (29)		17 (23)		.31
**Laboratory values or biomarkers**								
	Sodium (mEq/L), mean (SD)			139 (3)		139 (3)		.73
	Potassium (mEq/L), mean (SD)			4.26 (0.42)		4.27 (0.39)		.87
	Creatinine (mEq/L), mean (SD)			1.07 (0.59)		1.09 (0.33)		.81
	eGFR^i^ (mL/min/1.73 m^2^), mean (SD)			81 (24)		80 (21)		.80
	Blood urea nitrogen (mg/dL), mean (SD)			19.62 (8.72)		20.28 (8.33)		.55
	Glucose (mg/dL), mean (SD)			120 (53)		113 (34)		.23
	Hematocrit (%), mean (SD)			40 (4)		41 (5)		*.03*
	hsCRP^j^ (µg/mL), mean (SD)			6.89 (12.87)		9.10 (16.29)		.22
	NT-proBNP^k^ (ng/L), mean (SD)			2704 (6736)		2519 (1902)		.82
	IL-6^l^ (pg/mL), mean (SD)			3.05 (5.36)		4.32 (6.28)		.12
	IL-10^m^ (pg/mL), mean (SD)			10.74 (49.67)		13.67 (59.94)		.70
	TNF-αRII^n^ (pg/mL), mean (SD)			3425 (1700)		3456 (1671)		.90
	cTnT^o^ (ng/mL), mean (SD)			0.03 (0.08)		0.02 (0.05)		.62
	cTnI^p^ (ng/mL), mean (SD)			0.10 (0.28)		0.10 (0.25)		.98
	CK-MB^q^ (ng/mL), mean (SD)			3.94 (5.77)		3.87 (3.86)		.93
	Myoglobin (ng/mL), mean (SD)			31.37 (30.80)		37.13 (41.53)		.31
LVEF^r^: NonCMR^s^ LVEF (%), mean (SD)			24.2 (7.6)		23.0 (7.4)		.19	
**CMR structural and functional indices**								
	LVEF (%), mean (SD)			27.8 (10.3)		25.1 (8.8)		*.04*
	LV^t^ end-diastolic volume index (ml/m^2)^, mean (SD)			122.3 (39.9)		136.2 (48.4)		*.01*
	LV end-systolic volume index (ml/m^2^), mean (SD)			91.5 (39.1)		104.3 (45.2)		*.02*
	LV mass index (ml/m^2^), mean (SD)			75.1 (24.4)		80.3 (21.2)		*.09*
**CMR hyperenhancement**								
	LGE^u^ present (%), mean (SD)			176 (66)		56 (86)		*.002*
	Gray zone (g), mean (SD)			8.8 (11.6)		13.8 (12.2)		*.002*
	Core (g), mean (SD)			12.4 (14.9)		17.7 (15.1)		*.01*
	Total scar (g), mean (SD)			21.1 (25.4)		31.3 (25.6)		*.004*

^a^SCD: sudden cardiac death.

^b^*P* values <.05 are italicized.

^c^MI: myocardial infarction.

^d^NYHA: New York Heart Association.

^e^ACE: angiotensin-converting enzyme.

^f^ARB: angiotensin II receptor blocker.

^g^LBBB: left bundle branch block.

^h^ICD: implantable cardioverter defibrillator.

^i^eGFR: estimated glomerular filtration rate.

^j^hsCRP: high-sensitivity C-reactive protein.

^k^NT-proBNP: N-terminal pro-b-type natriuretic peptide.

^l^IL-6: interleukin-6.

^m^IL-10: interleukin-10.

^n^TNF-αRII: tumor necrosis factor alpha R II.

^o^cTnT: cardiac troponin T.

^p^cTnI: cardiac troponin I.

^q^CK-MB: creatine kinase MB.

^r^LVEF: left ventricular ejection fraction.

^s^CMR: cardiac magnetic resonance.

^t^LV: left ventricular.

^u^LGE: late gadolinium enhancement.

### Interpretability

**Figure 1:**
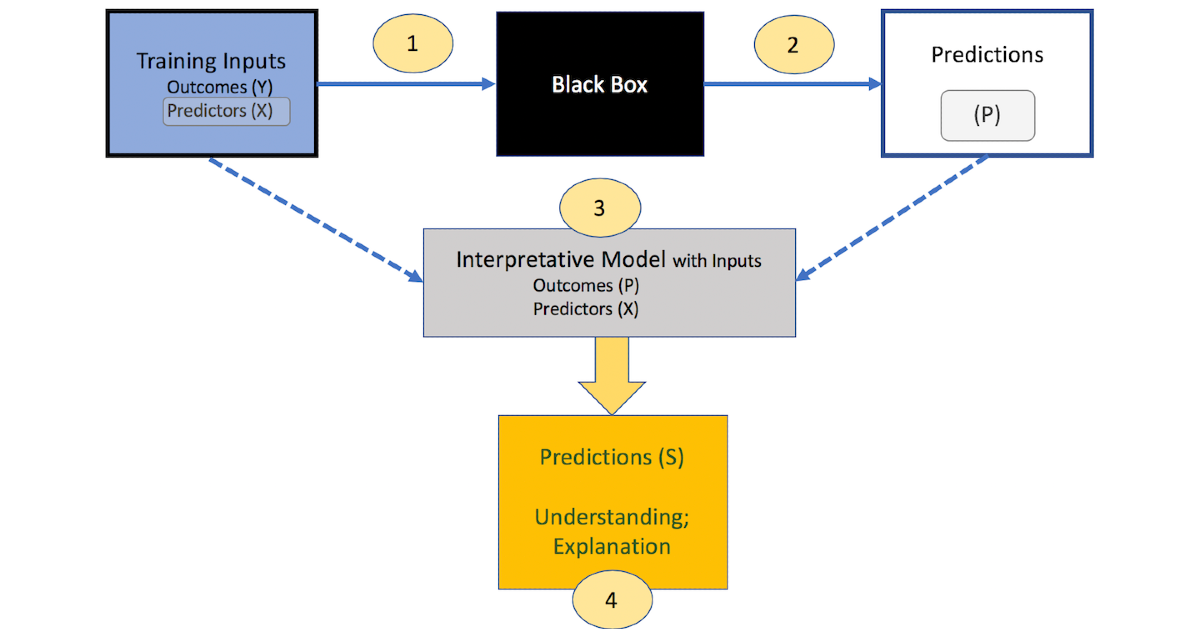
Steps to present machine learning (ML) predictions in an interpretable manner: The black box algorithm is applied to input data comprising outcomes (Y) and predictors (X) to obtain black-box predictions (P) of the input outcomes. The original X variables and the black-box predictions (P) are inputs to a simple model or algorithm, for example a single tree, whose predictions (S) are sufficiently close to (P) but more easily understood and explained.

**Figure 2:**
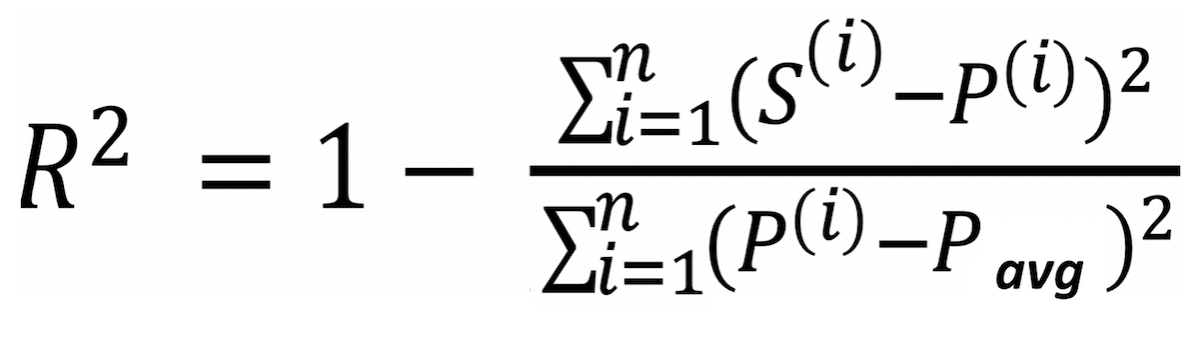
R^2^ equation where i=1 to n observations evaluated. S^(i)^ denotes the prediction for the i^th^ observation using the simplified model, P^(i)^ denotes the prediction for the ith observation using the ML model, and P_avg_ denotes the average prediction from the ML model.

To communicate the results from ML models, such as our RF for SCD predictions, we develop representative interpretable summaries. As illustrated in Figure 1, the following general steps can be employed to create simplified representations of any *black box* prediction:

1. Train the ML model with the input features (*X*) and the outcome of interest (*Y*).
2. Obtain the predicted values (*P*) from the ML model using cross-validation, a separate test dataset, or another data-division approach to ensure that predictions are not obtained from the same dataset used to train the model.
3. Train a simple, interpretable, and clinically understood model, such as a decision tree [34] or a linear or logistic regression model [35], using the predicted values (*P*) from the ML model as the outcome of interest and the corresponding input variables (*X*) from the original training dataset.
4. Obtain the predicted values (*S*) from the interpretive model. Calculate how close *S* is to *P*, that is how well the simplified model represents the ML model, using a measure such as R^2^, defined as provided in Figure 2.

Note that the interpretative tree can be grown sufficiently large such that R^2^ is arbitrarily close to 1. If a simple tree has a small R^2^, extra caution should be exercised to avoid overinterpreting the simplified model. In contrast, if R^2^ is high, the simplified model may be considered as an alternative to the actual ML model for obtaining future predictions in a simplified manner [36,37]. This model-agnostic approach to obtain a simplified summary of the ML model is shown in Figure 1.

By using a single tree as a summary of the RF predictions, we can quantify the importance of individual variables or groups of variables. A useful measure of the total effect on outcome *Y* of predictor (or group of predictors) *X_1_* is obtained by summing the improvements in prediction error (deviance) over all of the *X_1_* splits in the interpretative tree.

To present results in other ways familiar to clinicians, predictor effects can be communicated in plots where risk ratios are presented [38]. We created plots based on the relationship between the predictor variables and predicted risks. For categorical variables, risk ratios are calculated by comparing risks for different levels of the categorical variable (eg, risk ratio=[average predicted risk for males]/[average predicted risk for females] ). For continuous variables, risk ratios are calculated by comparing risks for different ranges of the continuous variable (eg, risk ratio=[average predicted risk for upper tertile of age]/[average predicted risk for lower tertile of age]). CIs for these risk ratios were generated through nonparametric bootstrap approaches [39]. All analyses were conducted using R 3.5.1 (R Foundation) [40].

## Results

### Global Summary Visualization

**Figure 3:**
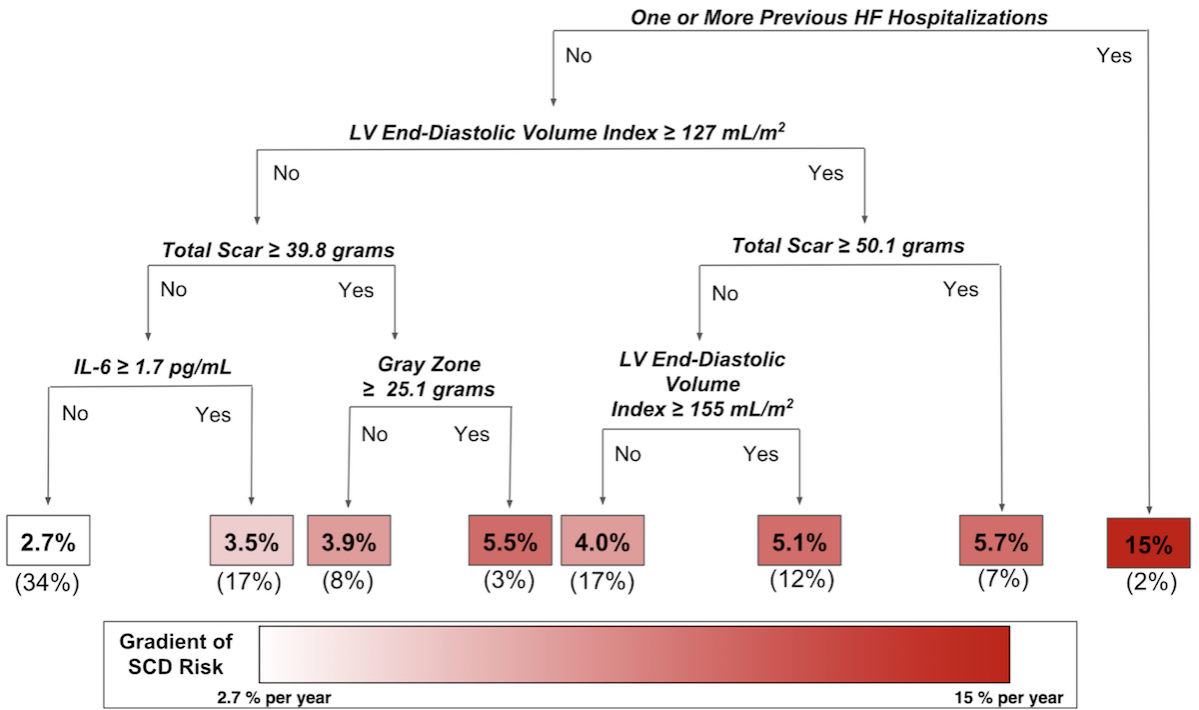
Global summary tree of random forest (RF) model for sudden cardiac death (SCD) prediction: Several risk factors (namely heart failure hospitalization, several cardiac magnetic resonance imaging indices, and interleukin-6 [IL-6], a marker of inflammation) discriminate between low-, intermediate-, and high- risk patients. Decision rules in the tree are shown in bold italics. The 1-year risks of SCD are shown in the boxes at the bottom of the decision tree. The boxes are colored according to the magnitude of the percent per year risk, with white corresponding to the lowest risk subgroup and dark red corresponding to the highest risk subgroup. Percentages in parentheses at the bottom of the boxes are the proportions of the total training data that belong to each of the risk subgroups. R^2^ is 0.88 for how well this global summary tree represents the RF model. HF: heart failure; LV: left ventricular.

Using data from the LV Structural Predictors of SCD Registry, a global summary for SCD risk prediction is obtained by fitting a single decision tree to RF predictions using as inputs the same covariates used in the RF and the outcome as the RF predictions. Figure 3 shows a global summary tree of the RF model for SCD prediction. Several risk factors appear as early split nodes in the decision tree representing key variables that discriminate between low -, intermediate -, and high-risk patients, including heart failure (HF) hospitalization history, CMR imaging indices (ie, LV end-diastolic volume index, and total scar and gray zone mass), and a measure of systemic inflammation, interleukin-6 (IL-6).

### Risk Ratio and Variable Importance

**Figure 4:**
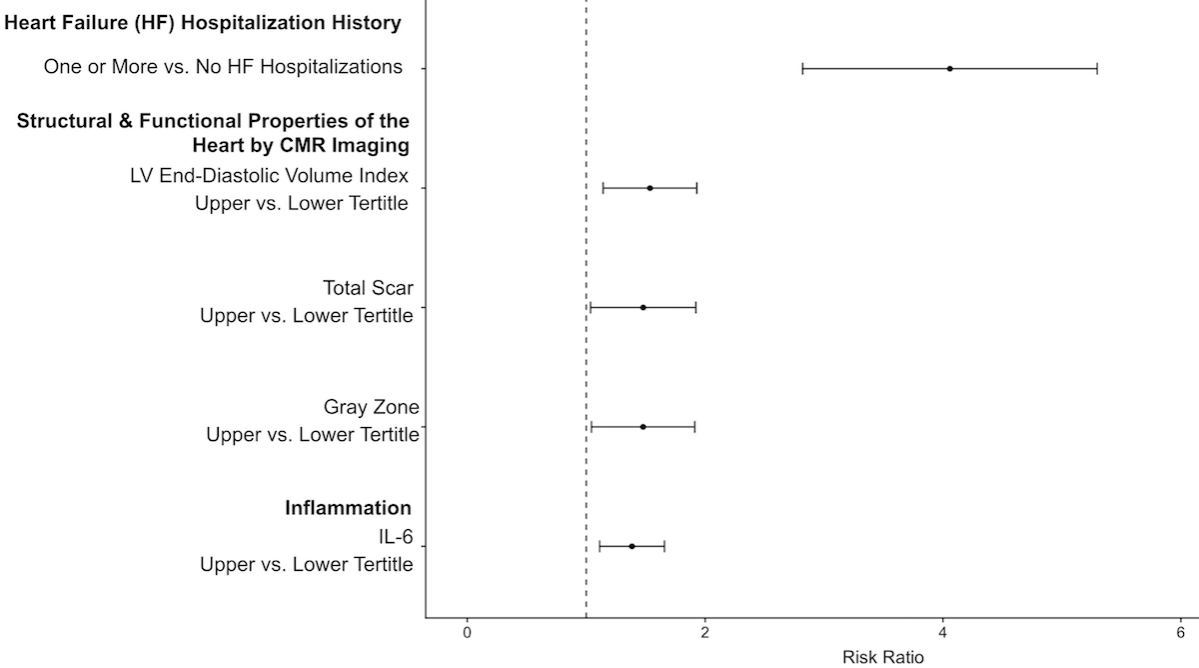
Visualization of predictor effects in random forest (RF) model for sudden cardiac death (SCD) prediction: Risk ratio point estimates and the 95% confidence intervals generated from 500 bootstrap replications are shown for the RF model for SCD risk prediction. The largest risk ratio is between individuals who never experienced a heart failure hospitalization and those who experienced one or more heart failure hospitalizations. The other risk ratio comparisons show the risk ratios between individuals grouped into different categories based upon inflammation or cardiac magnetic resonance (CMR) imaging variables indicating the structural and functional properties of the heart. HF: heart failure; IL-6: interleukin 6; LV: left ventricular.

Figure 4 shows the risk ratio plot for predictors identified as splitting variables in the global tree summary model for our RF SCD prediction example presented in Figure 3. The largest risk ratio is for HF hospitalization history before an arrhythmic event, indicating that individuals with 1 or more preceding HF hospitalizations are at 4.06 (95% CI 2.82-5.30) times higher risk of SCD than individuals without hospitalizations for HF. Comparing the risk for individuals in the upper versus lower tertile for CMR imaging variables and IL-6 demonstrates that higher values for these variables suggest a higher SCD risk. Specifically, the risk ratios were 1.54 (95% CI 1.14-1.93) for an LV end-diastolic volume index above 133 mL/m^2^ versus below 102 mL/m^2^; 1.48 (95% CI 1.04-1.92) for a total scar mass above 30.79 g versus below 1.48 g; 1.48 (95% CI 1.04-1.91) for a gray zone mass above 11.37 g versus below 0.40 g, and 1.38 (95% CI 1.11-1.66) for IL-6 above 2.15 pg/mL versus below 1.04 pg/mL.

Table 2 lists the predictor variables in their order of importance in the single interpretative tree shown in Figure 3. Their ranking is based on the fraction of total variation (deviance) in the ML predictions they explain in 1 or more splits in the single tree shown in Figure 3. Although there are only 8 terminal nodes in the tree, the tree explains 88% of the information in the predictions from the *black box* RF. Additionally, trees inherently identify interactions. Note that after the first split on whether or not a person had a prior hospitalization for HF, the imaging variable only predicted risk among persons without prior hospitalization. This asymmetry indicates that the absence of a prior HF hospitalization strongly interacts with the cardiac imaging variables.

**Table 2:** This table summarizes the global summary tree (shown in Figure 3) with an analysis of the variation (deviance) in the predicted values (P) from the machine learning (ML) model explained by the predictors in the global summary tree. The number of splits contributed by each variable in the global summary tree is enumerated along with the deviance and the percentage of the deviance explained. The predictors' ranked importance (ordered from most to least important from left to right in the table) is determined from the percentage of the deviance explained.

Split variable	HF^a^ hospitalization history	LV^b^ end-diastolic volume index	Total scar	Inflammation (IL-6^c^)	Gray zone	Tree total	ML^d^ total
Number of splits	1	2	2	1	1	7	N/A^e^
Deviance explained	1.26	0.255	0.100	0.034	0.020	1.67	1.89
Percentage of deviance explained	66.6	13.5	5.2	1.7	1.1	0.88^f^	100

^a^HF: heart failure.

^b^LV: left ventricular.

^c^IL-6: interleukin 6.

^d^ML: machine learning.

^e^N/A: not applicable.

^f^This corresponds to the R^2^ value (0.88) obtained when using the equation shown in Figure 3 for the calculations.

## Discussion

### Principal Findings

We demonstrate that it is possible to obtain improved transparency of ML by generating simplified models and visual displays adapted from those used commonly in clinical practice. As a specific example of this framework, we use RF extended to survival analysis with time-varying covariates for individualized SCD risk prediction. Commonly used methods for SCD risk prediction, such as Cox proportional hazards regression, do not automatically account for nonlinear and interaction effects or facilitate the application to individualized risk prediction [41,42]. In contrast to traditional regression strategies or parametric approaches that make assumptions about the underlying model, ML, such as RF, employs nonparametric algorithms that allow the data to *speak for themselves* and perform as powerful methods for individualized predictions [32,43-45]. RFs, as ensembles of decision trees, are not easily interpretable even though single decision trees are popular in medicine because of their intuitiveness and comparability to how a clinician tends to think through a case. The framework introduced in this work provides a methodology to increase ML transparency through representative interpretable summaries.

Because this framework for interpretability is model-agnostic, the user may benefit from ML’s high predictive performance while also gaining insights into how predictions were generated. Despite the complexity of the original algorithm, these methods for interpretability only depend on the *inputs* upon which the *black box* was trained and the corresponding *outputs* from the *black box*, namely its predictions. Thus, any method for prediction can be explained to an extent in a simplified manner. In a situation where it is not possible to capture the variation in ML predictions with a simple summary, the proposed method signals this problem through a natural comparison of the similarity between the predicted values from the ML and its approximating interpretative model. This approach extends prior research in mimic learning and *post hoc* explanations of the *black box* predictions [46,47]. This paper emphasizes the clinician’s perspective as the end user experienced with tree-based reasoning as a natural correlate of clinical reasoning.

To implement ML in clinical practice, it is essential to provide *user-centric* tools that allow clinicians to gain understanding and trust in their predictions [48]. Developing visualizations that are easy to interpret and based upon familiar ways clinicians understand algorithms or results can help communicate ML predictions. For example, simplifying RFs into a single decision tree produces a visualization that reflects medical treatment or diagnostic decision making in clinical practice. Although we illustrate the simplified model with a decision tree, other models such as linear regression can also be presented. Additionally, providing visual displays of risk ratio estimates in a manner similar to those presented in the medical literature may help clinicians gain an understanding of ML predictions.

Developing interpretable predictions is particularly important in the application of ML to health care because of the unique challenges related to medical ethics and regulatory or legal considerations [48]. Explanations that describe predictions can facilitate trust, especially when the explanations are consistent with domain knowledge or extend upon what is currently known [48]. For instance, in our illustrative example of SCD prediction in the LV Structural Predictors of SCD Registry, the key risk factors are HF hospitalization history, CMR imaging indices (ie, LV end-diastolic volume index, total scar, and gray zone mass), and a measure of inflammation (ie, IL-6). The predictors identified in our simplified summary are consistent with the published literature on SCD. It is known that among HF patients, SCD is a major cause of death due to complex interactions between the underlying myocardial substrate and triggers such as inflammation [22,49,50]. CMR imaging indices have been independently associated with ventricular arrhythmias in multiple cohorts [22,51-54]. This study raises the interaction hypothesis that cardiac imaging predictors are mainly useful in patients without prior HF hospitalizations. Visually seeing that predictions are grounded upon decision rules coinciding with clinical and biomedical knowledge (Figure 3) can help translate ML predictions for the end user’s understanding. Furthermore, presenting a summary visualization of the ML model along with information about the effect estimates of the predictors (Figure 4) can facilitate further insight.

### Limitations and Comparison With Prior Work

Although any complex model can be simplified to a summary model, it is possible that the summary and original model predictions are highly dissimilar, as reflected in a small R^2^. This was not the case in the motivating study, where 5 variables and 7 splits explained 88% of the variation in the RF’s predicted values. We can expect similar results in many problems because the interpretive tree is trained on the predicted values from a complex ML algorithm designed to find relatively lower-dimensional summaries than the original data. When a small interpretative tree has a poor R^2^, it can be enlarged as needed to achieve a prespecified higher value. The user can then look for simpler summaries by grouping classes of predictors and interactions among them. Finally, the approach has the R^2^ value as a measure of the fidelity of the simpler model predictions to the ML predictions. When this value is too small for a given tree, the user knows that a simple tree has limited interpretative value.

A closely related subfield of ML is actively addressing this topic by comparing different learning algorithms and selecting a final model [55]. Additionally, as the general approach for obtaining a simplified model summarizes the complex model at a global level, the simplified model is considered a global surrogate model and may not be representative of certain subgroups (eg, different subpopulations may exhibit different relationships between the predictor variables and predictions) [37]. To address this possibility, multiple simplified models could be created for each subgroup of interest. For example, two different summary decision trees could be created for men and women. Another area of active research is the development of local explanation models, where the interpretable models are local surrogate models that explain individual ML predictions rather than the entire *black box* as a whole [56]. Furthermore, although we emphasized here the tailoring of visual displays to clinicians, research in focus groups with both clinicians and patients can further accelerate the progress toward clinically meaningful ML developments that are translated into patient care.

### Conclusions

Currently, limited interpretability remains a major barrier to successful translation of ML predictions to the clinical domain [1-5]. Although numerous tools such as those for feature importance, feature effect, and prediction explanations have previously been developed to facilitate interpretability [9-12,56,57], the clinical community as a whole still generally considers ML as a field that generates *black box* predictions [1-5]. Although further research is necessary to fully understand the challenges limiting the clinical implementation of these tools, we believe that emphasis on tailoring explanations and visual displays to the end user is essential. Here, we expand upon the toolkit for opening the *black box* to the clinical community through the presentation of clinically relevant and interpretable visualizations to aid in the progress toward incorporating ML in health care. Ultimately, multidisciplinary teams with combined clinical and data science expertise are essential in furthering research to address the challenges limiting the clinical implementation of these powerful, informative ML tools.

## Abbreviations

CMR: cardiac magnetic resonance
HF: heart failure
IL-6: interleukin-6
LV: left ventricular
ML: machine learning
RF: random forest
SCD: sudden cardiac death
